# The Genetic Architecture of Shoot and Root Trait Divergence Between Mesic and Xeric Ecotypes of a Perennial Grass

**DOI:** 10.3389/fpls.2019.00366

**Published:** 2019-04-04

**Authors:** Albina Khasanova, John T. Lovell, Jason Bonnette, Xiaoyu Weng, Jerry Jenkins, Yuko Yoshinaga, Jeremy Schmutz, Thomas E. Juenger

**Affiliations:** ^1^Department of Integrative Biology, The University of Texas at Austin, Austin, TX, United States; ^2^Genome Sequencing Center, HudsonAlpha Institute for Biotechnology, Huntsville, AL, United States; ^3^United States Department of Energy, Joint Genome Institute, Walnut Creek, CA, United States

**Keywords:** adaptation, ecotype, genetic architecture, quantitative trait locus, root architecture, pleiotropy, epistasis, recombinant inbred line

## Abstract

Environmental heterogeneity can drive patterns of functional trait variation and lead to the formation of locally adapted ecotypes. Plant ecotypes are often differentiated by suites of correlated root and shoot traits that share common genetic, developmental, and physiological relationships. For instance, although plant water loss is largely governed by shoot systems, root systems determine water access and constrain shoot water status. To evaluate the genetic basis of root and shoot trait divergence, we developed a recombinant inbred population derived from mesic and xeric ecotypes of the perennial grass *Panicum hallii*. Our study sheds light on the genetic architecture underlying the relationships between root and shoot traits. We identified several genomic “hotspots” which control suites of correlated root and shoot traits, thus indicating genetic coordination between plant organ systems in the process of ecotypic divergence. Genomic regions of colocalized quantitative trait locus (QTL) for the majority of shoot and root growth related traits were independent of colocalized QTL for shoot and root resource acquisition traits. The allelic effects of individual QTL underscore ecological specialization for drought adaptation between ecotype*s* and reveal possible hybrid breakdown through epistatic interactions. These results have implications for understanding the factors constraining or facilitating local adaptation in plants.

## Introduction

Adaptations to abiotic stress have been implicated as driving factors in ecological speciation (Stebbins, 1952; Lexer and Fay, 2005), where populations have diverged across a number of traits, exhibit different niche characteristics, and eventually become reproductively isolated (Clausen, 1951; Lowry, 2012; Yardeni et al., 2016). Local adaptation to soil water availability is an especially important driver of plant evolution (Stebbins, 1952; Rajakaruna, 2004; Kooyers et al., 2015) and can impose strong natural selection on populations, leading to the formation of ecotypes that are differentially adapted to xeric and mesic habitats (Joly et al., 1989; Kumar et al., 2008). Xeric and mesic ecotypes are often characterized by the divergence of common suites of morphological and phenological traits (Clausen, 1951; Lowry, 2012) related to maintaining water status and tolerating drought (Chapin et al., 1993; Markesteijn and Poorter, 2009; Juenger, 2013).

While leaf and shoot traits are important drivers of adaptation to drought (Carmo-Silva et al., 2009; Juenger, 2013), the properties of root systems determine plant water access and can place constraints on shoot water status (Price et al., 2002; Hund et al., 2009). Shoot traits may be related to root traits through genetic correlation (Bouteillé et al., 2012) or be dependent upon root traits through resource allocation tradeoffs (Hammer et al., 2009), including changes in carbon allocation between root and shoot systems (Hummel et al., 2010). Higher root mass ratio (RMR) increases water foraging capability to maintain plant water status, which can be accomplished by allocating more resources toward roots (Knight et al., 2006) or by inhibiting above ground growth (Hendriks et al., 2015). Specific leaf area (SLA, the ratio of leaf area to leaf dry mass) and specific root length (SRL, the ratio of root length to root dry mass) are both important plant traits linked to resource acquisition (Reich, 2014; Cheng et al., 2016) and SRL is typically thought of as the below ground analog of SLA (Eissenstat et al., 2000; Reich, 2014). These traits are often positively correlated (Withington et al., 2006; Reich, 2014; Valverde-Barrantes et al., 2017) and associated with rapid growth (Pérez-Harguindeguy et al., 2013; Reich, 2014) – where an acquisitive root strategy (high SRL) can be aided by an acquisitive leaf strategy (high SLA; Pérez-Ramos et al., 2013). Despite evidence that root and shoot trait covariance is an important driver of plant adaptation, few studies have documented how combinations of specific shoot and root traits generate locally adapted ecotypes. The genetic basis of such trait complexes and the implications of recombining adaptive shoot and root traits in hybrids are poorly understood.

Quantitative genetic analyses and the mapping of quantitative trait loci (QTL) permit exploration of the genetic basis of trait correlations and trait divergence (Fishman et al., 2002; Lovell et al., 2015; Milano et al., 2016). Importantly, by simultaneously analyzing multiple traits, QTL mapping can infer the loci and genetic interactions that drive ecological trait correlations. Functional traits with a high degree of correlation that underlie divergence can result from pleiotropy through shared developmental genetics or genetic linkage (Via and Hawthorne, 2005; Lovell et al., 2013) as a result of correlational selection (Brodie et al., 1995). For example, colocalized QTL for root and shoot traits including root biomass, root volume, shoot biomass and plant height have been identified in a wheat recombinant inbred line (RIL) population (Iannucci et al., 2017) likely resulting from pleiotropy or tightly physically linked genes. Overall, there is growing evidence for substantial genetic variation in root system architecture and root/shoot relationships. However, the loci driving these trait correlations and the degree to which these patterns impact plant productivity are largely unknown.

*Panicum hallii* is a small, self-fertilizing, C_4_ perennial bunch grass native to North America that occurs across a large geographical range comprised of diverse habitats and climates. Average annual precipitation ranges from 127 cm per year on the eastern border of its distribution to 13 cm per year on the west. *P. hallii* occurs as two distinct ecotypes (xeric upland and mesic lowland) that are classified as separate varieties, *P. hallii* var. *hallii* (hereafter referred to as *hallii*) and *P. hallii* var. *filipes* (hereafter referred to as *filipes*). *Hallii* is typically found in xeric upland habitats with shallow, dry, calcareous and rocky soils in the American southwest and northern Mexico; while *filipes* occurs in mesic lowland areas on clay and silt soils mostly along the Gulf Coast Plain of Texas and Mexico (Gould, 1975; Waller, 1976). The xeric upland ecotype, *hallii*, is smaller in stature and overall size than the mesic lowland ecotype *filipes*: with smaller leaves, fewer tillers, earlier flowering time, fewer flowers per inflorescence, but larger seed size and seed mass (Waller, 1976; Lowry et al., 2013). This is consistent with its polyploid relative, *Panicum virgatum* (an important biofuel candidate), where upland ecotypes are typically smaller, flower earlier (Lowry et al., 2014a) and have less leaf area (McMillan, 1965) than lowland ecotypes. Previous analyses of shoot traits in a F_2_ population of *P. hallii* (Lowry et al., 2014b) demonstrated that a few large-effect loci drove multivariate shoot trait divergence between *hallii* and *filipes*, and complete genomes has been assembled and compared (Lovell et al., 2018). Here, we investigate the genetic architecture of multidimensional root phenotypic traits and their relationship with shoots to develop a more complete picture of the adaptive differences between these ecotypes.

In this study, we cross xeric and mesic ecotypes of *P. hallii*, to generate a population of RIL at the F_7_ generation and subsequently constructed a new genetic map based on whole genome re-sequencing. We utilized extensive phenotyping of root and shoot traits and a quantitative genetic approach to identify the genetic architecture of trait relationships and their divergence among ecotypes. We discovered shared QTL clusters involved in genetic correlations between root and shoot growth related traits that were independent of QTL clusters for carbon allocation and phenology related traits. The allelic effects of individual QTL underscore ecological specialization for drought adaptation between *hallii* and *filipes* and reveal possible hybrid breakdown through epistatic interactions.

## Materials and Methods

### Morphological Shoot and Root Phenotyping Under Greenhouse Conditions

We developed a population of RILs derived from a cross of *hallii* and *filipes* and constructed a genetic map from whole genome re-sequencing (see Supplementary Appendix 1). Raw sequence data was deposited in the NCBI short read archive (see Supplementary Table 5). Seedlings of 174 F_7_ RILs and the two parental genotypes were planted to 6 × 30 cm Cone-Tainers (Stuewe and Sons, Tangent, OR, United States) filled with Field and Fairway Profile (The Turf Trade, NJ, United States) media. Plants were grown in a completely randomized block design within three blocks on a single bench at the University of Texas greenhouse (see Supplementary Appendix 2). Plants were harvested within three days of a common developmental stage defined as when a fully expanded flag leaf with a visible ligule was observable on any tiller with an emerging panicle. Harvest dates across the population ranged from 27 to 51 days after germination. The tiller height, leaf length and area of the flag leaf of the main tiller were measured and tiller number was counted at the time of harvest. Total root number was counted and then the root system was spread out in a clear acrylic water filled tray and scanned at a 600 dpi resolution using an EPSON Scanner (Model 12000XL, Epson America, Inc., San Jose, CA, United States) calibrated for use with WinRhizo Pro 2015 root image analysis software (Regent Instruments Inc., Canada). Leaf, shoot and root tissue was dried and weighed to obtain biomass. SLA was calculated for each plant as the ratio of leaf area to dry mass (Supplementary Appendix 2).

Root trait data was obtained from scans using WinRhizo Pro 2015 software and included total root length (cm), total root volume (cm^3^), and average root diameter (mm). Specific root length (SRL; cm g^-1^), root tissue density (RTD, g cm^-3^), and RMR were calculated for each plant (Supplementary Appendix 2).

### Data and QTL Analysis

Data analyses centered on fitting linear mixed models and considered RIL genotype as a fixed effect (proc mixed, SAS) for the measured phenotypic traits. Block was also included as a fixed effect covariate when it had a significant impact on measured traits (emergence day, specific root length and root diameter). The SAS procedure PROC CORR was used to calculate genetic correlation coefficients of traits based on RIL line means. Broad-sense trait heritability was calculated using h2boot software using one-way ANOVA among inbred RILs with 1000 bootstrap runs (Phillips and Arnold, 1999). Trait divergence between parental lines was evaluated with a *t*-test in SAS.

The majority of the measured traits were continuously distributed with relatively strong multivariate structure based on pairwise correlational analyses. As such, we also used genetic principal component analysis (PCA) to obtain a multidimensional overview of shoot and root trait variation and integration. PCA was performed on the trait means of each line for the following phenotypic variables: emergence day, tiller number, root number, root biomass, shoot biomass, root diameter, root tissue density, specific root length, specific leaf area, tiller height, leaf length, root volume and total root length. PCA was completed using SAS with the proc princomp function. The first three principal components that together explained 75% of total variation were retained for QTL analysis.

Quantitative trait locus mapping was completed in R using the R/qtl package (Broman and Sen, 2009) on the RIL breeding values as described above (Supplementary Table 1). When quantitative trait data distributions were not normally distributed, data was log (emergence day, tiller number) or square root (shoot biomass) transformed. Two functions were used to determine the position of QTL and to conduct the calculation of estimates for additive effects and epistasis (an additive-by-additive interaction between quantitative trait loci) (script^1^). The scantwo function with 1000 permutations was used to calculate penalties for main effect and interactions for each phenotypic trait, and the stepwise QTL function was used to conduct a forward-backward search and account for epistasis with a maximum of 6 QTL (at least two QTL peaks in addition to those detected with the scanone function) that optimized the penalized LOD score criterion. Threshold values for type 1 error rates were set at alpha = 0.05 for all traits based on permutation. 1.5 LOD drop intervals of QTL were calculated using the qtlStats function (Lovell, 2018). In addition, QTL analysis was performed on the first three principal components following the above procedure.

### Confirming Root and Shoot Biomass QTL in a Field Study

To further confirm and evaluate major QTL detected in our greenhouse study, we conducted a follow up field experiment on a focal QTL during the 2016 growing season. Ten RILs homozygous at the shared QTL region for root and shoot biomass were selected for this experiment (5 with *filipes* alleles and 5 with *hallii* alleles). Eight biological replicates of each selected RIL line and eight replicates of the two parental genotypes were planted on May 10, 2016 under both restrictive and well-watered irrigation treatments [(10 RILs + 2 parents) × 8 biological replicates × 2 irrigation levels = 192 plants; see Supplementary Appendix 2]. Plants were harvested toward the end of the summer growing season in August. Shoots were separated from roots, dried at 55°C for 4 days before weighing for biomass. Trait values more extreme than 1.5 × the interquartile range were removed as outliers prior to analysis. For statistical analysis, we used linear mixed models with proc mixed in SAS. The main effect for the model was genotype at the focal QTL (*filipes* or *hallii* alleles at the marker position), treatment and genotype-by-treatment interaction. RIL line was used as a random effect to control for background genetic variance.

## Results

### Heritable Shoot and Root Trait Differences Between Mesic and Xeric Ecotypes

The RIL parents representing mesic and xeric ecotypes of *Panicum hallii* (HAL2 and FIL2) had significantly different shoot and root trait mean values (Table 1). The xeric genotype, HAL2, had 2.3-fold earlier first panicle emergence (*t* values at 5 dfs and *P* values; *t* = 2.87, *P* = 0.035), 3.3-fold less shoot biomass (*t* = 4.39, *P* = 0.007) and 2.8-fold less root biomass (*t* = 3.08, *P* = 0.028), 1.8-fold shorter plant height (*t =* 3.43, *P* = 0.018), 2.2-fold shorter leaf length (*t* = 6.3, *P* = 0.001), 2-fold shorter total root length (*t* = 3.29, *P* = 0.022), 2.5-fold lower total root volume (*t* = 3.41, *P* = 0.02), and 1.3-fold increased specific root length (*t* = -2.5, *P* = 0.05) relative to the mesic genotype FIL2 (Table 1).

**Table 1:** FIL2 and HAL2 root and shoot trait value means with SE and *t*-statistics; and RIL root and shoot trait value means, range, and broad-sense heritability (*H*^2^) with SE.

Phenotypic Trait	FIL2	HAL2	*t*	*P-*value	RIL mean	RIL range	*H*^2^±SE
Panicle Emergence (day)	9.25 1.19	4.00 1.38	2.87	**0.035**	7.01 1.74	1.00–18.33	0.51 0.05
Shoot Biomass (g)	4.74 0.49	1.41 0.57	4.39	**0.007**	1.65 0.33	0.29–4.74	0.59 0.05
Tiller Number (count)	6.25 0.48	5.00 0.56	1.68	0.150	6.00 0.83	3.00–14.50	0.50 0.05
SLA (cm^2^g^-1^)	325.62 18.15	382.77 20.96	–2.06	0.094	381.58 33.17	264.67–499.36	0.18 0.08
Plant Height (cm)	21.18 1.82	11.63 2.11	3.43	**0.018**	12.57 1.56	4.30–23.65	0.63 0.04
Leaf Length (cm)	30.77 1.72	14.23 1.98	6.30	**0.001**	15.66 1.46	4.75–24.27	0.66 0.04
Root Biomass (g)	1.38 0.18	0.51 0.21	3.08	**0.028**	0.54 0.10	0.12–1.60	0.58 0.06
Root Number (count)	14.00 0.97	8.33 1.11	3.84	**0.012**	8.87 1.39	2.50–15.00	0.38 0.05
SRL (cm g^-1^)	10.14 0.85	13.37 0.98	–2.50	0.055	12.27 1.11	6.12–17.95	0.43 0.06
RTD (g cm^-3^)	0.06 0.01	0.05 0.01	1.31	0.247	0.05 0.01	0.03–0.08	0.39 0.07
Root Diameter (mm)	0.46 0.01	0.44 0.02	1.27	0.259	0.45 0.01	0.37–0.55	0.37 0.05
Root Volume (cm^3^)	2.43 0.28	0.98 0.32	3.41	**0.019**	1.00 0.17	0.26–2.90	0.56 0.05
Root Length (m)	1.37 0.14	0.67 0.16	3.29	**0.022**	0.65 0.11	0.12–1.64	0.59 0.04
RMR (ratio)	0.22 0.01	0.27 0.01	–3.44	**0.018**	0.25 0.02	0.16–0.39	0.34 0.09

*t-statistics given at 5 degrees of freedom with statistically significant *P-*values indicated in bold text.*

We estimated broad-sense trait heritability (*H*^2^) as the proportion of observed phenotypic variance due to genetic differences among RILs in the population. In the RIL population, all measured traits were heritable, with *H*^2^ ranging from 18 to 66% for shoot traits and from 34 to 60% for root traits (bootstrap based significance, in all cases *P* < 0.001). The most heritable traits were leaf length (66%), plant height (64%), shoot biomass (60%), root length (60%) and root biomass (58%; Table 1). Transgressive segregation, where the range of recombinant phenotypes extends beyond the range of parental values (Rieseberg et al., 1999), was found among the majority of traits except shoot biomass, plant height, leaf length, root biomass and root number, where FIL2 had trait values that were the highest or close to the highest of population wide values, while HAL2 values were generally in the middle of the population trait distribution (Table 1).

Many shoot and root phenotypic traits showed remarkably strong genetic correlations in the RIL population (Supplementary Table 2). For example, shoot and root biomass (*r* = 0.92, *P* < 0.0001), tiller and root number (*r* = 0.67, *P* < 0.001), shoot biomass and root volume (*r* = 0.91, *P* < 0.0001), and shoot biomass and total root length (*r* = 0.90, *P* < 0.001) were all positively genetically correlated. We performed PCA to characterize the multivariate structure of our data. The first three PCA axes explained 75% of the overall trait variance. Principal component one (PC1; 45.5% variance explained) was composed of general plant size traits (shoot biomass, root biomass, number of tillers, number of roots, tiller height, leaf length, root volume and root length). Principal component two (PC2; 16.5%) was mainly composed of root resource acquisition traits (SRL, root diameter and root tissue density). Principal component three (PC3; 12.6%) was composed of carbon acquisition and allocation traits (SLA, RMR and panicle emergence; Supplementary Table 3 and Supplementary Figure 1).

### QTL Underscore Root and Shoot Trait Divergence Between *Hallii* and *Filipes*

**FIGURE 1:**
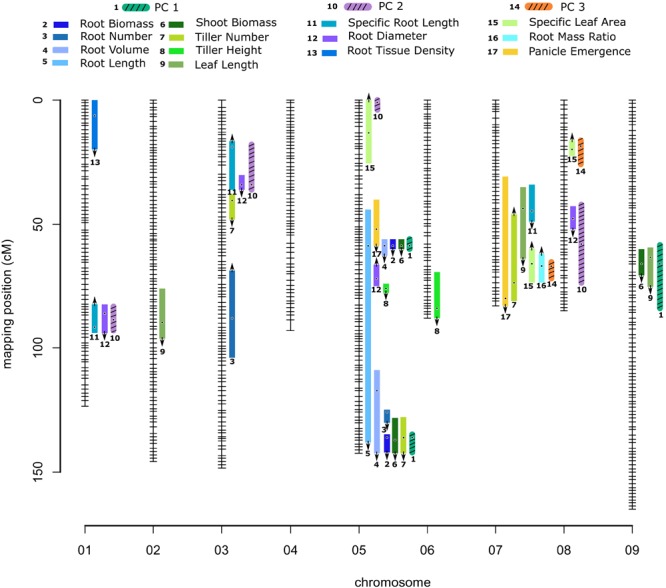
Genetic map of the *Panicum hallii* RIL population with location of trait QTL. Colored bars indicate 1.5-LOD drop confidence intervals. Location of dots within the bars is the location of QTL peaks. Arrow represents the direction of additive effect, with up or down arrows indicating that the *hallii* allele increases or decreases the trait value.

Given high *H*^2^ values, it is not surprising that QTL were detected for all measured traits. A total of 32 QTL were identified for 14 phenotypic traits: two QTL for one phenological trait, 14 QTL for five shoot traits and 16 QTL for eight root traits (Table 2, Figure 1, and Supplementary Figure 2). QTL for all traits showed additive effects in the direction of parental divergence, except for one of three QTL for tiller number, one of four QTL for root diameter, and one of three QTL for SRL. *Filipes* alleles had later panicle emergence and increased trait values for plant size related traits, including: emergence day, root number, root tissue density, root biomass, shoot biomass, tiller height, leaf length and root volume. *Hallii* alleles increased trait values associated with water acquisition (SRL) and carbon acquisition and allocation (RMR, SLA).

**Table 2:** Main and epistatic effects of QTL for the *Panicum hallii* RIL population.

Phenotype	Chr	Peak (cM)	1.5 Lod Interval	LOD	% var	Effect	SE	Positive allele donor	QTL Cluster (CL)
Panicle Emergence	5	52.1	40–59	4.59	9.85	–0.044	0.009	*filipes*	CL5.1
(day)	7	80.0	31–83	4.31	9.2	–0.039	0.008	*filipes*	CL7.2
Shoot Biomass	5	58.6	56–60	7.43	14.8	–0.044	0.007	*filipes*	CL5.1
(g)	5	136.0	128–142	5.08	9.82	–0.031	0.007	*filipes*	CL5.3
	9	66.1	60–71	4.78	9.19	–0.027	0.005	*filipes*	CL9.1
	Epi5:5			2.86	5.36	0.027	0.007		
Tiller Number	3	40.5	38–48	7.23	14.74	–0.054	0.009	*filipes*	
(count)	5	137.0	128–142	3.47	6.73	–0.037	0.009	*filipes*	CL5.3
	7	73.6	46–81	4.84	9.56	0.039	0.008	*hallii*	CL7.2
SLA	5	13.3	0–26	3.15	5.25	9.772	2.543	*hallii*	
(cm^2^g^-1^)	7	66.0	60–74	8.56	15.37	16.394	2.494	*hallii*	CL7.2
	8	19.8	16–23	8.33	14.90	16.077	2.484	*hallii*	
Tiller Height	5	76.0	74–77	6.16	13.34	–1.765	0.320	*filipes*	CL5.2
(cm)	6	83.9	69–88	3.82	8.05	–1.096	0.256	*filipes*	
Leaf Length	2	89.7	76–96	4.28	8.56	–1.19	0.264	*filipes*	
(cm)	7	43.6	35–64	4.39	8.80	–1.293	0.283	*filipes*	CL7.1
	9	63.4	59–75	3.41	6.76	–0.985	0.246	*filipes*	CL9.1
Root Biomass	5	58.6	56–60	8.81	18.61	–0.012	0.002	*filipes*	CL5.1
(g)	5	136.0	135–142	8	16.71	–0.010	0.002	*filipes*	CL5.3
	Epi5:5			4.61	9.21	0.008	0.002		
Root Number	3	88.0	69–104	6.18	13.9	–1.08	0.199	*filipes*	
(count)	5	125.7	125–130	5.36	11.94	–0.81	0.196	*filipes*	CL5.3
	Epi3:5			2.79	5.99	0.73	0.202		
SRL (cm g^-1^)	1	91.5	82–94	5.3	11.02	0.66	0.131	*hallii*	CL1.1
	3	18.8	17–36	5.16	10.7	0.78	0.156	*hallii*	CL3.1
	7	44.7	34–49	3.16	6.4	–0.55	0.145	*filipes*	CL7.1
RTD (g cm^-3^)	1	6.3	0–20	3.15	7.9	–0.001	0.0004	*filipes*	
Root Diameter	1	86.0	82–94	4.73	8.68	–0.009	0.002	*filipes*	CL1.1
(mm)	3	34.2	30–36	5.36	9.91	–0.011	0.002	*filipes*	CL3.1
	5	71.9	66–75	3.78	6.84	0.010	0.002	*hallii*	CL5.2
	8	47.9	43–52	4.65	8.50	–0.009	0.002	*filipes*	
Root Volume	5	58.6	56–63	3.96	8.85	–0.134	0.030	*filipes*	CL5.1
(cm^3^)	5	117.2	109–142	3.07	6.77	–0.119	0.032	*filipes*	CL5.3
Root Length (cm)	5	58.6	44–138	3.12	7.85	–0.82	21.29	*filipes*	CL5.1,2,3
RMR (ratio)	7	67.0	62–74	6.36	15.34	0.0137	0.002	*hallii*	CL7.2

*Chr, chromosome; Peak, cM (centimorgan) position of the QTL peak; LOD, logarithm of odds; % var, percent of variance; SE, one standard error; SLA, specific leaf area; SRL, specific root length; RTD, root tissue density; RMR, root mass ratio; Epi, epistasis.*

The additive effects of each QTL explained from 5.25 to 15.4% of phenotype variation for shoot traits, and from 5.9 to 18.6% for root traits (Table 2). Of these 32 QTL, eight QTL occupied unique positions in the genome: root tissue density on chr1, leaf length on chr2, tiller number on chr3, root number on chr3, SLA on chr5 and chr8, tiller height on chr6, and root diameter on chr8. As expected, three of these single QTL were also identified by principle component QTL (Supplementary Table 4 and Figure 1). The confidence intervals of all other QTL are shared or colocalized with at least one other QTL.

### Trait-Specific QTL Cluster Into Genomic “Hotspots”

We identified three major and five minor clusters of root and shoot trait QTL occurring over five different chromosomes (Table 2 and Figure 1). Here we identify QTL clusters (CL) by chromosome and numerical order from the telomere for each chromosome. As expected, we found that positions of QTL for principle components were highly indicative of the locations of QTL clusters for the traits loading on particular PC axes (Supplementary Table 4, Figure 1, and Supplementary Figure 2).

Quantitative trait locus for PC1 localized to three genomic clusters of QTL for plant size traits. CL9.1 contains shoot biomass and leaf length QTL. CL5.1 contains root biomass, shoot biomass, root volume, total root length and panicle emergence QTL. CL5.3 contains root biomass, shoot biomass, root volume, total root length, tiller number and root number QTL. A separate QTL pair for tiller height and root diameter not identified with PC1 lies between these two large clusters. PC2 QTL localized with one of two genomic clusters of QTL for root resource acquisition traits. CL1.1 and 3.1 both contain SRL and root diameter traits. PC3 QTL localized to a single genomic cluster (CL7.2) related to carbon allocation traits. CL7.2 contains panicle emergence day, leaf length, number of tillers, RMR and SLA. Near this PC3 associated QTL is a minor cluster (CL7.1) of leaf length and SRL (Table 2, Supplementary Table 4, and Figure 1).

**FIGURE 2:**
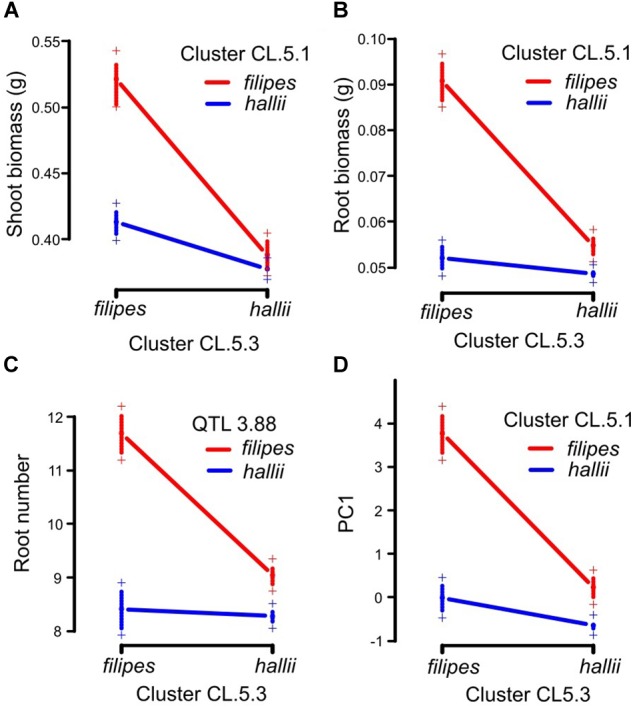
Pairwise epistatic QTL in the *P. hallii* RIL population. Plotted points indicate two-locus genotype means ± 1SE for the two loci containing root biomass between CL.5.1 and CL.5.3 **(A)**, shoot biomass between CL.5.1 and CL.5.3 **(B)**, root number between QTL 3.88 and CL.5.3 **(C)** and PC1 between CL.5.1 and CL5.3 **(D)**.

Four pairwise epistatic interactions, where the effect of one QTL depends on the allelic state of an unlinked QTL, were detected (Table 2, Supplementary Table 4, and Figure 2). Three QTL from cluster CL5.3 (shoot biomass, root biomass and PC1) interacted with other QTL for these traits located in CL5.1. In addition, the root number QTL from CL5.3 interacted with the root number QTL on chr3. Individuals that possess the *hallii* allele for these QTL at CL5.3 mask the positive effects of their interactive QTL.

### A Major Pleotropic Effect QTL Is Confirmed in the Field

**FIGURE 3:**
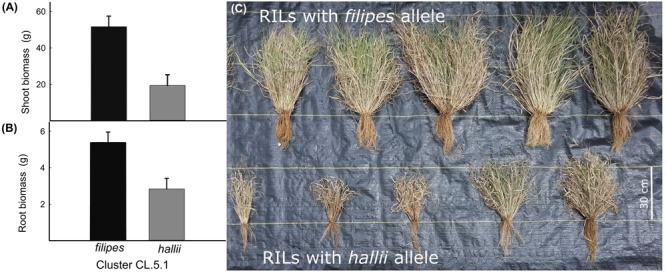
Mean ± 1SE of shoot biomass **(A)** and root biomass **(B)** for field grown *P. hallii* RILs homozygous for either *filipes* or *hallii* parental alleles at shoot and root biomass QTL located in cluster CL5.1. Picture of field grown RILs homozygous at CL5.1 for *filipes* allele (top row) and *hallii* allele (bottom row) **(C)**.

To confirm the effects of QTL observed in a controlled greenhouse study, we phenotyped two sets of RILs homozygous for different parental alleles at the loci for shoot and root biomass (CL5.2) in a field experiment. While the magnitude of increased biomass for lines with *filipes* alleles at the selected QTL observed in the field is 24% less for the root biomass and 11% less for the shoot biomass relative to the greenhouse, the effects are significant and in the same direction as those observed in the greenhouse. Field grown lines with *filipes* parental alleles produced 1.9-fold more root biomass (*P* = 0.0024) and 2.7-fold more shoot biomass (*P* = 0.0002) relative to field grown lines with *hallii* parental alleles (Figure 3). In addition, the HAL2 parental line showed a 1.8-fold increase trend in RMR (*P* = 0.09) over the FIL2 parental line under field conditions compared to the 1.2-fold difference observed in the greenhouse (*P* = 0.018). There were no significant differences between the irrigation treatments or the interaction of treatment by genotype for RILs or the parental genotypes. However, root biomass showed a 1.2-fold increase trend under the dry treatment relative to the wet treatment (*P* = 0.08).

## Discussion

Ecotypes are often differentiated by suites of correlated root and shoot traits that may share common genetic and developmental architectures as a result of adaptive differentiation. One of our major findings was several genomic “hotspots” of colocalized QTL for multiple shoot and root traits. This is consistent with a previous study of a *P. hallii* F_2_ population covering a suite of ecotype differentiating shoot trait QTL which clustered on chr5 (Lowry et al., 2014b). In addition to confirming this important locus, we discovered additional root traits linked to this region along with additional regions of clustered loci for root and shoot traits. Colocalized QTL controlling traits such as root biomass, shoot biomass, among others, has also been shown in RIL populations of wheat and sorghum (Mace et al., 2012; Iannucci et al., 2017). These findings indicate that specific loci can shape both shoot and root morphological traits, through tight linkage of several genes controlling individual traits or a single pleiotropic gene that controls several traits.

PC1 QTL localized to three genomic regions controlling several size related root and shoot traits (shoot biomass, root biomass, root volume, and other). We found that the *hallii* allele had additive effects in the direction of ecotype divergence and contributed to smaller root and shoot phenotypes in every case compared to the *filipes* allele. This finding is consistent with the global pattern observed in angiosperm plants whose shoot and root biomass are positively correlated (Enquist and Niklas, 2002) and with other studies on perennial grasses where total biomass is decreased under water limited conditions (Baruch, 1994; Weißhuhn et al., 2011; Tozer et al., 2017). Importantly, we show that one of the main growth QTL effects is robust to the environment and persists under natural field conditions.

In addition to differences in absolute size, there are expected differences in carbon acquisition and allocation between xeric and mesic ecotypes. PC3 resulted from cluster of carbon allocation and phenology related traits (SLA, RMR, tiller number, and panicle emergence). Plants with *hallii* alleles had greater SLA, RMR, tiller number, and faster panicle emergence. Thinner leaves (high SLA) have lower carbon cost and are associated with increased photosynthetic capacity (Reich et al., 1997; Cornelissen et al., 2003). Increased RMR helps to maintain plant water status and productivity under drought (Comas et al., 2013). Faster flowering time along with greater tiller number allows for rapid production of seeds when resources are available for short time periods. These factors combined may indicate that *hallii* employs a fast acquisitive strategy for drought escape; acquiring nutrients rapidly and flowering quickly to enter a dormant state before periods of summer drought. Acquisitive shoot and root strategies have been associated with fast growth strategies and summer dormancy in other perennial grasses (Balachowski et al., 2016). This contrasts with the lower SLA, and RMR of the mesic *filipes*, which may employ a slow strategy of thicker longer lasting leaves, larger more persistent roots, and abundant above ground foliage. This common genetic control of ecotype differentiating traits involving shoot and root organs suggests that these factors evolved in tandem. Alternatively, we found a relatively weak genetic correlation between SLA and SRL, which are important plant traits linked to resource acquisition (Reich, 2014; Cheng et al., 2016) and associated with fast growth (Pérez-Harguindeguy et al., 2013; Reich, 2014). Each of these traits had three independent QTL. Thus, divergence of these traits is likely due to independent loci which become structured across ecotypes as a result of strong directional or correlational selection. In this case, our crossing scheme was able to largely decouple these traits through recombination.

Observed pairwise epistatic interactions for root biomass, shoot biomass and root number showed that *hallii* alleles mask the effects of *filipes* alleles in all cases. When lines are homozygous for *hallii* parental alleles at CL5.3, it contributes to smaller phenotypes for these traits, regardless of the genotype at their respective interactive QTL. This suggests that the CL5.3 loci could include a pleiotropic gene with major effect that controls the development of multiple shoot and root size related traits. Natural populations of *P. hallii* ecotypes are largely homozygous, thus these linked QTL likely work together in a positive direction and contribute to the phenotypic trait correlations that underlie ecotype divergence. The observed epistasis in the RIL population could be involved in ecological speciation (Burke and Arnold, 2001), and these interactions in hybrid plants could be deleterious and impact survivorship by undermining synergistic trait relationships. For example, the combination of reduced root and shoot size effected by *hallii* alleles may be desirable in xeric environments, but deleterious in natural hybrids or under the higher competition mesic environments that *filipes* inhabits.

### Greenhouse Detected Genetic Correlations Confirmed Under Field Conditions

There is persistent concern that effects observed in greenhouse studies are not representative of plant performance in natural or agronomic environments. Although greenhouse and growth chambers may be able to replicate a wide range of temperature and light conditions, other differences between these artificial and natural environments can be significant. Furthermore, greenhouse studies are often conducted on very young plants and in smaller than optimal pots, which can significantly alter root architectures compared to natural environments. Several recent studies have highlighted how differences in conditions between glasshouse and natural settings can affect the mapping of genetic architectures for various plant traits (Poorter et al., 2012; reviewed in Lovell et al., 2016).

We sought to overcome this concern by confirming the glasshouse detected genetic architecture of two of our chief traits of interest (root biomass and shoot biomass) in selected RILs and parental genotypes in a field setting at full plant maturity. In the RILs, we found that our glasshouse observed QTL were confirmed. For the parental lines, we found that RMR differences between the xeric and mesic ecotypes nearly doubled under field conditions as compared to the glasshouse study. This suggests that adaptive allocation of biomass to roots increases with plant age and can also be constrained by pot limitations in the glasshouse. More importantly, these results provide credence to the assumption that our glasshouse study is predictive of plant performance in a natural setting. Future studies with *P. hallii* should explore the genetic architecture of shoot:root traits over multiple perennial seasons in additional field studies. These data will help to clarify the lifetime fitness consequences of allocation strategies and potential ecological tradeoffs that arise in natural habitats.

## Conclusion

In the process of ecotype formation, populations can diverge across many functional traits and exhibit different niche characteristics, which requires coordination between plant organ systems. Root traits are involved in adaptive differentiation to abiotic stresses by their direct effects on water acquisition, and through correlation, tradeoffs or constraints with shoot traits (Hammer et al., 2009; Mace et al., 2012). Our study sheds light on the genetic architecture underlying the relationships between root and shoot traits involved in ecotype divergence of *P. hallii* and demonstrates that some correlated traits are under common genetic control as a result of QTL colocalization and interaction, while other traits are controlled by independent loci. We found several genomic hotspots relating to multiple root and shoot traits and a striking pattern of epistatic interaction impacting overall plant growth. Further insight into the molecular basis of these loci will be an important step in understanding the genetic coordination and ecological importance of root and shoot systems involved in ecotype divergence.

## Data Availability

The raw sequencing data was deposited at NCBI, phenotypic data is included as Supplementary Material.

## Author Contributions

AK and TJ designed the experiments. AK and JB conducted the experiments. JJ and JS conducted the *Panicum hallii* genome assembly. XW, YY, JJ, and JS performed sequencing of the RIL population. JL created the genomic map. AK, JL, JB, and TJ contributed to statistical analysis. AK wrote the first draft of the manuscript, JL, JB, and TJ contributed to revisions.

## Conflict of Interest Statement

The authors declare that the research was conducted in the absence of any commercial or financial relationships that could be construed as a potential conflict of interest.

## Footnotes

1 https://github.com/AlbinaKh/P.hallii_RIL_RootShoot_QTLmapping
