# Supplementary material for: The Genetic Architecture of Shoot and Root Trait Divergence Between Mesic and Xeric Ecotypes of a Perennial Grass
Source: Front Plant Sci. 2019 Apr 4;10:366. doi: 10.3389/fpls.2019.00366 (PMC6458277; doi:10.3389/fpls.2019.00366)
Supplement: Supplementary file 1 [file Data_Sheet_1.docx]

**Supplementary Appendix 1.**

**Development of the RIL mapping population**

We developed a population of recombinant inbred lines (RILs) in order to evaluate the genetic basis of divergence between *hallii* and *filipes*. The parents of the RIL mapping population were genotypes selected from populations of the upland and lowland ecotypes of *P. hallii*. The upland parent (HAL2-11, hereafter referred to as HAL2) was a one-generation selfed progeny of an individual selected from a glasshouse planting of seed collected from a natural population of *hallii* located at the Lady Bird Johnson Wildflower Center (Austin, TX, USA; 30.16°N, 97.87°W). The lowland parent (FIL2) was selected from a glasshouse planting of seed collected from a natural population of *filipes* located near the coastal city of Corpus Christi, Texas (27.65°N, 97.40°W). FIL2 and HAL2 represent the genome reference genotypes for *filipes* and *hallii* respectively (https://phytozome.jgi.doe.gov/pz/portal.html#!info?alias=Org_Phallii) and are largely homozygous individuals. A cross of these two genotypes, with HAL2 as the maternal parent, yielded an F_1_ hybrid and self-fertilized seed obtained from this individual was used to establish a large F_2_ population (Lowry, 2012). A number of these F_2_ progeny were selected at random and propagated repeatedly via single seed descent until the F_6_ generation. DNA was obtained from leaf tissue of F_6_ seedlings and submitted for whole genome resequencing at the DOE Joint Genome Institute through the Community Science Program. F_7_ seed was subsequently collected from the sequenced F_6_ individuals for this experiment.

SNPs were called from whole genome resequencing of 356 RILs on four Illumina 2x150 runs at 12x coverage. Libraries were quality filtered using the fastx toolkit ‘fastq_quality_filter’ program with a quality threshold of 33. Filtered reads were mapped to a soft masked *P. hallii* reference genome (FIL-2 V2.0) using bwa mem with the default parameters. Mapped reads were filtered by samtools –Shb with a quality of 20. Bam files were indexed, sorted and duplicates were removed with picard. Reads adjacent to insertions / deletions were masked using GATK RealignerTargetCreator and reads were re-sorted and re-indexed prior to SNP calling. SNPs were called via GATK haplotypeCaller independently for each library, producing a gVCF for each. These were merged and re-genotyped by GATK’s genotypeGVCF and condensed into a 0/1/2 (alternate allele counts) matrix with vcfTools. Genotype data from 335 RILs were included in the output genotype matrix. The resultant matrix was processed in R. SNPs with >10% and <80% homozygotes and <5% NA and <20% heterozygotes were retained.

We applied a 3-step sliding window approach for marker calling: 1) The genome was broken into 200 marker windows (overlapping by 100 markers) and the proportion of each genotype was calculated. 2) Training data was constructed, retaining the 100 strongest heterozygous sites and a random sampling of 100 of the sites with > the mean proportion of each homozygote; 3) A random forest machine learning model was fit to the training data (the R caret package) and used to predict the genotypes of all sliding window intervals resulting in a 3361 marker matrix. Raw sequence data was deposited in the NCBI short read archive under the BioProject ID in Supplementary Table 5.

**Genetic map construction**

To build the genetic map, we culled the genotype matrix such that no two markers could have a pairwise recombination fraction <0.005. This culling procedure minimized the amount of segregation distortion and missing data within any 0.5 cM window. Linkage groups were formed from the resulting 1278 marker matrix. Markers were ordered within linkage groups using a travelling salesperson problem solver as implemented through the concorde program and parsed through the TSPMap function tspOrder (Monroe et al*.*, 2017). We then fine-tuned the resulting genetic map first by culling the genotype matrix to a 711-marker grid where no markers resided <1cM from an adjacent marker, then looking at improving the fine-order of markers using the ripple algorithm. Finally, chromosomes were named and oriented to maximize the similarity with the physical position of markers in the FIL2 genome annotation (phytozome.net).

**Reference**

Lowry, D.B. (2012). Ecotypes and the controversy over stages in the formation of new species. *Biol. J. Linn. Soc.* 106, 241–257. doi:10.1111/j.1095-8312.2012.01867.x.

Monroe, J.G., Allen, Z., Tanger, P., Mullen, J.L., Lovell, J.T., Moyers, B.T., *et al.* (2017). TSPmap: A tool making use of traveling salesperson problem solvers in the efficient and accurate construction of high-density genetic linkage maps. *BioData Mining*, 10, 38.
