# Supplementary material for: The Genetic Architecture of Shoot and Root Trait Divergence Between Mesic and Xeric Ecotypes of a Perennial Grass
Source: Front Plant Sci. 2019 Apr 4;10:366. doi: 10.3389/fpls.2019.00366 (PMC6458277; doi:10.3389/fpls.2019.00366)
Supplement: Supplementary file 2 [file Data_Sheet_2.docx]

**Supplementary Appendix 2.**

**Greenhouse experiment**

Seeds of 174 F_7_ RILs and the two parental genotypes were scarified with sandpaper and placed on wet sand in round petri dishes on September 5, 2016 and allowed to germinate in a greenhouse located at the University of Texas at Austin, Brackenridge Field Lab (12-h days at 500 μE m ^−2^ s ^−1^, 28°C; 12-h nights at 24°C). On the 7^th^ day after sowing, seedlings were transferred to 6 cm x 30 cm Cone-Tainers (Stuewe and Sons, Tangent, OR). Cone-Tainers were lined with 1 mil plastic liners (perforated at the bottom for drainage) to facilitate separation of the plant and root systems from the container during harvest. Cone-tainers were filled with Field and Fairway Profile (The Turf Trade, NJ, USA) media. Plants were then assigned to a completely randomized block design within three blocks on a single greenhouse bench. Plants were bottom watered by block by soaking to saturation every three days with Grow liquid nutrient solution (DynaGro, Richmond, CA) to promote seedling growth. Plants were harvested within three days of a common developmental stage defined as when a fully expanded flag leaf with a visible ligule was observable on any tiller with an emerging panicle. The plant in its plastic bag was pulled from the pot gently to prevent damage to the root system. Then the bag was cut open and the profile substrate was gently removed by shaking the plant on wire mesh followed by light washing of the root system in a bucket of tap water. Shoot material was separated from root material. The tiller height (from base of the plant to the node of the flag leaf on the tiller with the emergent panicle), leaf length and area of the flag leaf of the main tiller were measured and tiller number was counted at the time of harvest. Total root number was counted and then the root system was spread out in a clear acrylic water filled tray and scanned at a 600 dpi resolution using an EPSON Scanner (Model 12000XL, Epson America, Inc., San Jose, CA, USA) calibrated for use with WinRhizo Pro 2015 root image analysis software (Regent Instruments Inc., Canada). The Lagarde’s local threshold parameter in the analysis software was enabled to ensure detection of thin and pale roots and the diameter class size was set to 0.25 mm. Root trait data was obtained from scans using WinRhizo Pro 2015 software and included total root length (cm), total root volume (cm^3^), and average root diameter (mm). Leaf, shoot and root tissue was collected separately, dried for 96 hours in an oven at 55°C, and weighed to obtain biomass.

Specific root length (SRL; total root length / root biomass (cm g^-1^)), root tissue density (RTD; root biomass / total root volume (g cm^-3^)), root mass ratio (RMR, root biomass / total biomass) and specific leaf area (SLA; fresh leaf area / dry mass of the leaf (cm^2^ g^-1^)) were calculated for each plant.

**Confirming root and shoot biomass QTL in a field study**

Seed of selected lines were germinated and established in the greenhouse using the procedure outlined above for the RIL planting and subsequently transplanted into the field at the age of one month. Eight biological replicates of each line and eight replicates of the parental genotypes were planted on May 10, 2016 under both restrictive and well-watered irrigation treatments ((10 RILs + 2 parents) x 8 biological replicates x 2 irrigation levels = 192 plants).

The field experiment was conducted at a site located within the Brackenridge Field Laboratory property of the University of Texas in Austin, TX, USA (N 30.2845, W 97.7809). The site elevation is 133 m above sea level and soils are Yazoo sandy loam greater than 1.2 m deep. The mean maximum temperature (August) is ~35.0 °C and the mean minimum temperature (January) is ~ 3.0 °C. This experiment was co-planted in vacant space within an existing *P. hallii* experiment which was established at a site capable of providing two separate levels of irrigation. The site contains 32 differentially irrigated ‘beds’ which are separated underground by 1.2-meter-deep plastic sheeting (Regal Plastics, Austin, TX, USA) to prevent the spread of applied irrigation water. Irrigation was applied by dripline (0.9 GPH, 12” emitter spacing, Rain Bird, Azusa, CA). The treatment period occurred from June through August with the restrictive treatment receiving 4.5 fold less irrigation in both number of irrigation events and total amount of water applied.

Plants were harvested towards the end of the summer growing season in August over a three-day period. To account for differences in size of the plants, an equal volume of the soil under each plant was harvested using a ‘shovelomics’ device that regulated shovel angle and depth while extracting plants from the field soil. Plants with roots attached were rinsed clean of soil over a metal screen. Shoots were separated from roots, dried at 55°C for 4 days before weighing for biomass.
