# Supplementary material for: The Genetic Architecture of Shoot and Root Trait Divergence Between Mesic and Xeric Ecotypes of a Perennial Grass
Source: Front Plant Sci. 2019 Apr 4;10:366. doi: 10.3389/fpls.2019.00366 (PMC6458277; doi:10.3389/fpls.2019.00366)

Supplementary Figure 1. Principal component analysis of shoot and root traits for the *Panicum hallii* RIL population. Traits: PC, principal component; RMR, root mass ratio; SLA, specific leaf area; SRL, specific root length; RTLRNGTH, root length; LFLG, leaf length; HEIGHT, plant height; SHMASS, shoot biomass; RTMASS, root biomass; RTVOL, root volume; RTN, root number; TN, tiller number; RTD, root tissue density; ED, emergence day; RTDM, root diameter.

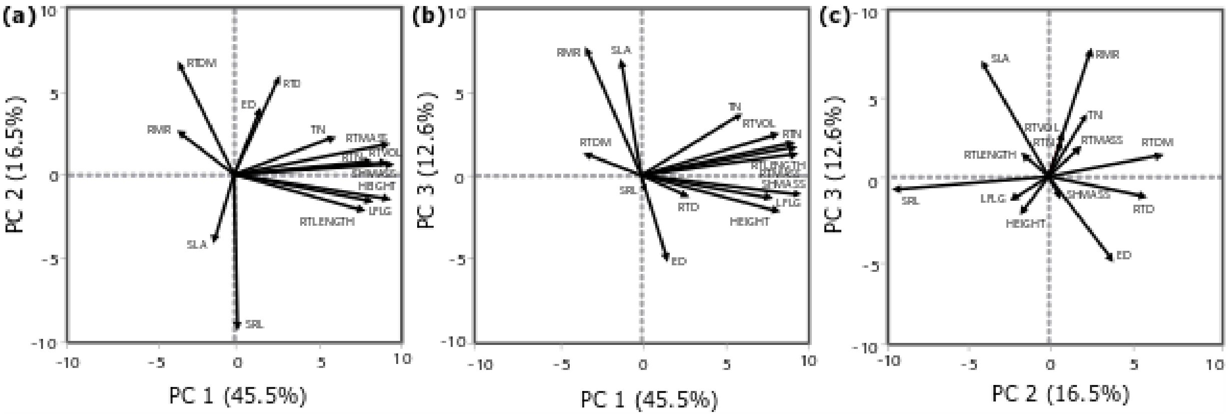

Supplement: Supplementary file 3 [file Image_1.pdf]
