# Supplementary material for: The Genetic Architecture of Shoot and Root Trait Divergence Between Mesic and Xeric Ecotypes of a Perennial Grass
Source: Front Plant Sci. 2019 Apr 4;10:366. doi: 10.3389/fpls.2019.00366 (PMC6458277; doi:10.3389/fpls.2019.00366)
Supplement: Supplementary file 4 [file Image_2.pdf]

**Supplementary Figure 2 Conditional** LOD profile plots of detected QTL for shoot and root traits and first three principle components of a *P. hallii* RIL mapping population resulting from the final model of stepwise QTL mapping.

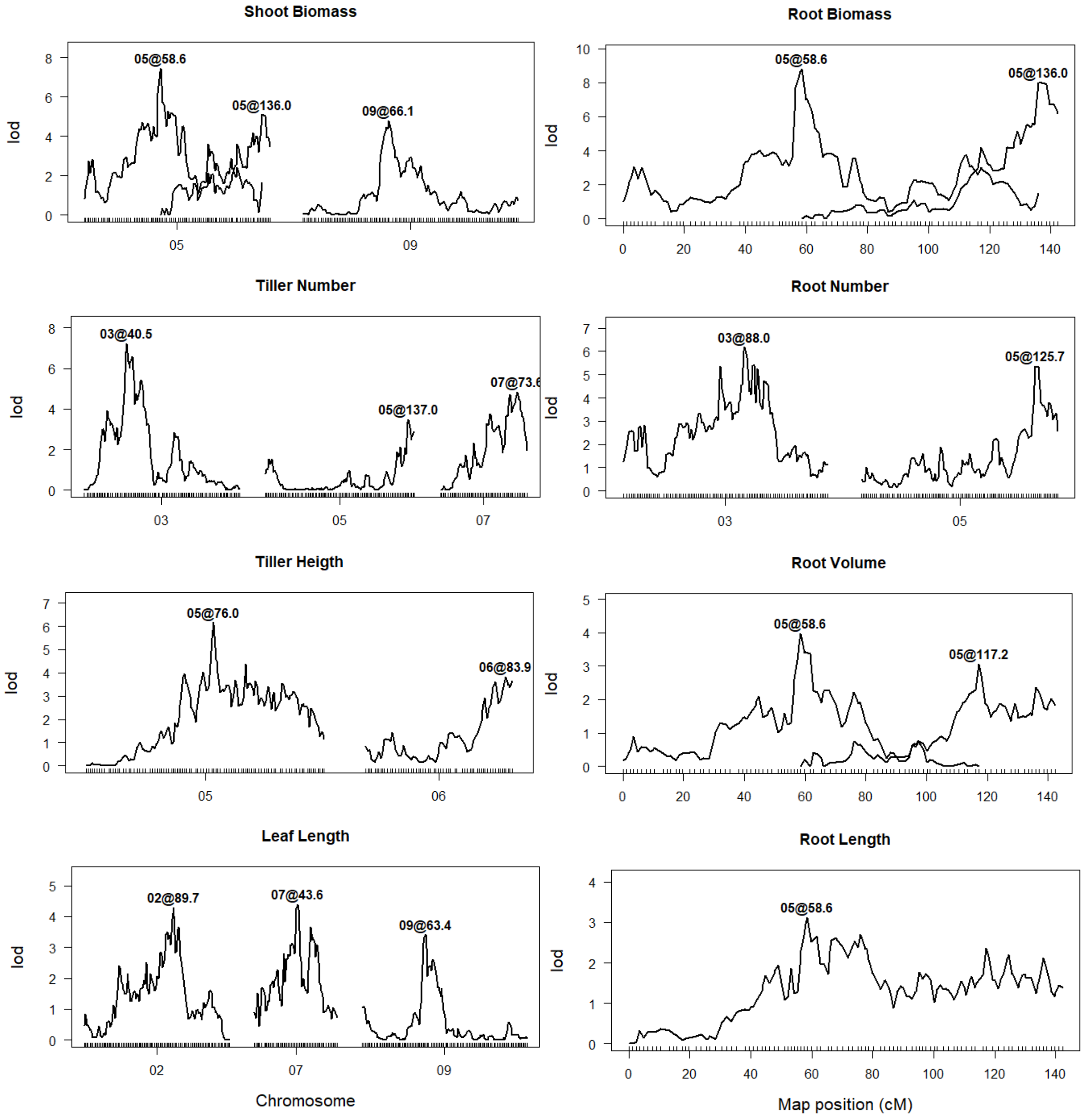

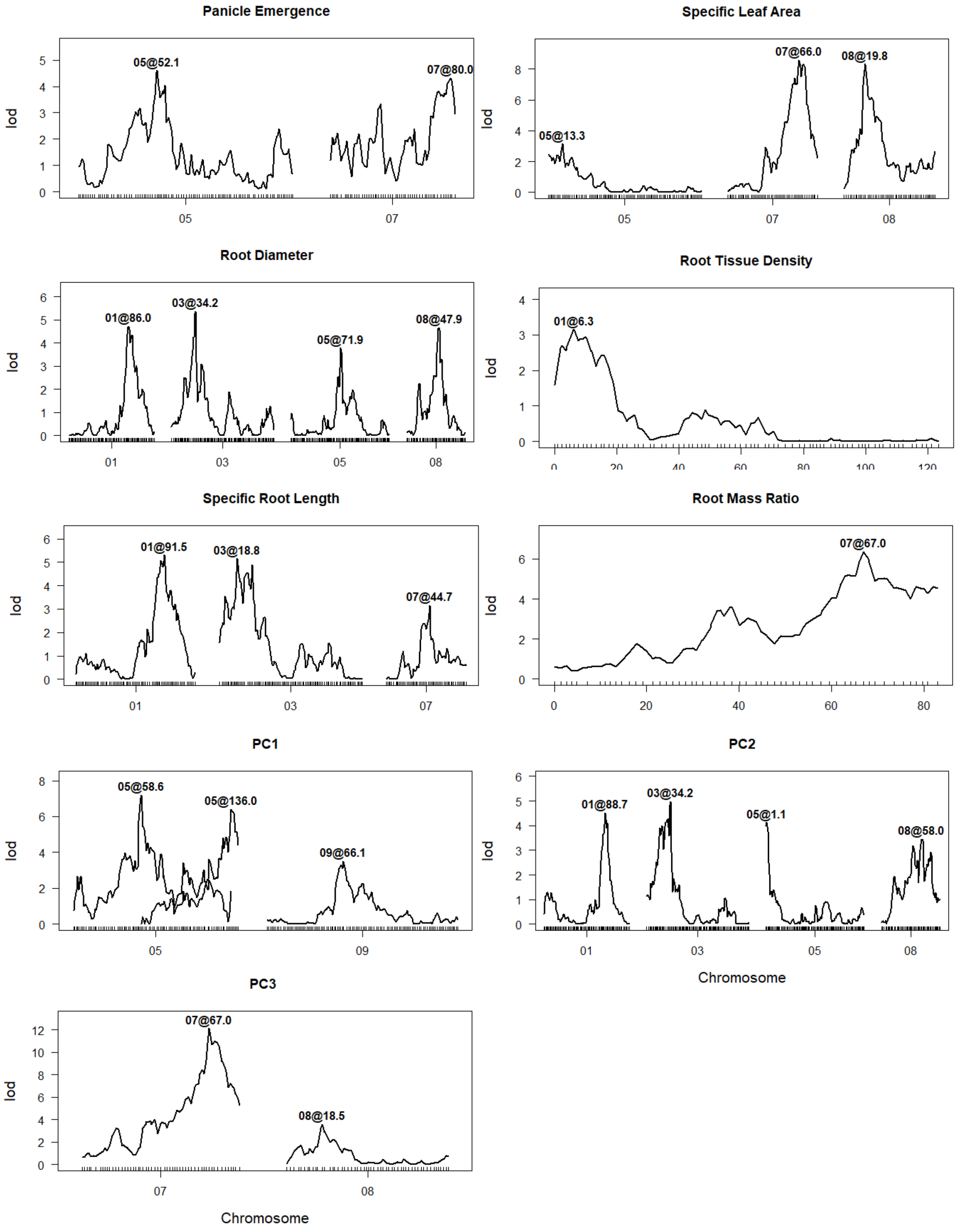
Panicle Emergence
05@52.1
07@80.0
lod
05
07
Specific Leaf Area
05@13.3
07@66.0
08@19.8
lod
05
07
08
Root Diameter
01@86.0
03@34.2
05@71.9
08@47.9
lod
01
03
05
08
Root Tissue Density
01@6.3
lod
0
20
40
60
80
100
120
Specific Root Length
01@91.5
03@18.8
07@44.7
lod
01
03
07
Root Mass Ratio
07@67.0
lod
0
20
40
60
80
PC1
05@58.6
05@136.0
09@66.1
lod
05
09
PC2
01@88.7
03@34.2
05@1.1
08@58.0
lod
01
03
05
08
Chromosome
PC3
07@67.0
08@18.5
lod
07
08
Chromosome
