# Supplementary material for: The Genetic Architecture of Shoot and Root Trait Divergence Between Mesic and Xeric Ecotypes of a Perennial Grass
Source: Front Plant Sci. 2019 Apr 4;10:366. doi: 10.3389/fpls.2019.00366 (PMC6458277; doi:10.3389/fpls.2019.00366)
Supplement: Supplementary file 6 [file Table_2.docx]

**Supplementary Table 2.** Pearson Correlation Coefficients for genetic correlations in the *Panicum hallii* RIL population.

| **Trait** | **ED** | **TN** | **RTN** | **SHMASS** | **RTMASS** | **SRL** | **RTD** | **HEIGHT** | **LFLG** | **RMR** | **SLA** | **RTDM** | **RTLG** |
| --- | --- | --- | --- | --- | --- | --- | --- | --- | --- | --- | --- | --- | --- |
| **TN** | 0.116 |  |  |  |  |  |  |  |  |  |  |  |  |
| **RTN** | 0.039 | **0.669** |  |  |  |  |  |  |  |  |  |  |  |
| **SHMASS** | **0.215** | **0.545** | **0.758** |  |  |  |  |  |  |  |  |  |  |
| **RTMASS** | 0.132 | **0.615** | **0.789** | **0.921** |  |  |  |  |  |  |  |  |  |
| **SRL** | **-0.192** | **-0.115** | -0.05 | -0.021 | **-0.15** |  |  |  |  |  |  |  |  |
| **RTD** | **0.195** | **0.195** | **0.191** | **0.281** | **0.336** | **-0.544** |  |  |  |  |  |  |  |
| **HEIGHT** | 0.119 | **0.285** | **0.598** | **0.824** | **0.719** | 0.118 | 0.136 |  |  |  |  |  |  |
| **LFLG** | 0.015 | **0.2** | **0.573** | **0.759** | **0.678** | 0.135 | 0.109 | **0.769** |  |  |  |  |  |
| **RMR** | **-0.281** | -0.005 | -0.121 | **-0.424** | -0.085 | **-0.267** | 0.029 | **-0.495** | **-0.399** |  |  |  |  |
| **SLA** | **-0.331** | 0.079 | 0.001 | -0.223 | -0.095 | **0.259** | **-0.288** | -0.114 | -0.047 | **0.388** |  |  |  |
| **RTDM** | 0.125 | -0.071 | **-0.183** | **-0.260** | **-0.163** | **-0.696** | -0.115 | **-0.317** | **-0.338** | **0.290** | -0.135 |  |  |
| **RTLG** | 0.057 | **0.566** | **0.772** | **0.905** | **0.925** | **0.185** | **0.198** | **0.762** | **0.734** | **-0.196** | 0.003 | **-0.450** |  |
| **RTVOL** | 0.120 | **0.614** | **0.802** | **0.911** | **0.975** | -0.059 | **0.150** | **0.722** | **0.670** | -0.107 | -0.045 | -0.134 | **0.934** |
| ED, panicle emergence; TN, tiller number; RTN, root number; SHMASS, shoot biomass; RTMASS, root biomass; SRL, specific root length; RTD, root tissue density; HEIGHT, plant height; LFLG, leaf length; RMR, root mass ratio; SLA, specific leaf area; RTDM, root diameter; RTLG, root length. Significant correlations are indicated in bold text. | | | | | | | | | | | | | |
