# Supplementary material for: The Genetic Architecture of Shoot and Root Trait Divergence Between Mesic and Xeric Ecotypes of a Perennial Grass
Source: Front Plant Sci. 2019 Apr 4;10:366. doi: 10.3389/fpls.2019.00366 (PMC6458277; doi:10.3389/fpls.2019.00366)
Supplement: Supplementary file 7 [file Table_3.docx]

**Supplementary Table 3.** Principal component (PC) loadings of measured traits in the *Panicum hallii* RIL population.

| **Trait** | **PC1** | **PC2** | **PC3** |
| --- | --- | --- | --- |
| Panicle Emergence (day) | 0.158 | 0.378 | -0.510 |
| Shoot Biomass (g) | 0.970 | 0.065 | -0.118 |
| Tiller Number (count) | 0.607 | 0.215 | 0.373 |
| SLA (cm^2^ g^-1^) | -0.119 | -0.423 | 0.699 |
| Plant Height (cm) | 0.840 | -0.153 | -0.220 |
| Leaf Length (cm) | 0.793 | -0.224 | -0.137 |
| Root Biomass (g) | 0.943 | 0.191 | 0.174 |
| Root Number (count) | 0.836 | 0.082 | 0.252 |
| SRL (cm g^-1^) | 0.039 | -0.951 | -0.080 |
| RTD (g cm^-3^) | 0.273 | 0.563 | -0.139 |
| Root Diameter (mm) | -0.337 | 0.707 | 0.131 |
| Root Volume (cm^3^) | 0.937 | 0.101 | 0.202 |
| Root Length (m) | 0.954 | -0.136 | 0.132 |
| RMR (ratio) | -0.336 | 0.263 | 0.773 |

SLA, specific leaf area; SRL, specific root length; RTD, root tissue density; RMR, root mass ratio.
