# Supplementary material for: The Genetic Architecture of Shoot and Root Trait Divergence Between Mesic and Xeric Ecotypes of a Perennial Grass
Source: Front Plant Sci. 2019 Apr 4;10:366. doi: 10.3389/fpls.2019.00366 (PMC6458277; doi:10.3389/fpls.2019.00366)
Supplement: Supplementary file 8 [file Table_4.docx]

**Supplementary Table 4.** Main and epistatic effects of the first three principal component QTL for the *Panicum hallii* RIL population.

| **Principal Component** | **Chr** | **Peak (cM)** | **1.5 Lod Interval** | **LOD** | **% var** | **Effect** | **SE** | **Donor of Positive allele** | **QTL Cluster (CL)** |
| --- | --- | --- | --- | --- | --- | --- | --- | --- | --- |
| PC1 | 5 | 58.6 | 56-60 | 7.19 | 14.42 | -1.215 | 0.209 | *filipes* | CL5.1 |
|  | 5 | 136.0 | 135-142 | 6.34 | 12.67 | -1.049 | 0.206 | *filipes* | CL5.3 |
|  | 9 | 66.1 | 58-84 | 3.51 | 6.7 | -0.664 | 0.164 | *filipes* | CL9.1 |
|  | Epi5:5 |  |  | 3.14 | 6.0 | 0.812 | 0.212 |  |  |
| PC2 | 1 | 88.7 | 83-93 | 4.51 | 8.26 | -0.458 | 0.099 | *filipes* | CL1.1 |
|  | 3 | 34.2 | 18-36 | 4.97 | 9.14 | -0.533 | 0.109 | *filipes* | CL3.1 |
|  | 5 | 1.1 | 0-4 | 4.13 | 7.52 | 0.457 | 0.103 | *filipes* |  |
|  | 8 | 58.0 | 42-74 | 3.45 | 6.24 | 0.392 | 0.097 | *filipes* |  |
| PC3 | 7 | 67.0 | 65-72 | 12.14 | 25.05 | 0.676 | 0.084 | *hallii* | CL7.2 |
|  | 8 | 18.5 | 16-26 | 3.59 | 6.60 | 0.354 | 0.085 | *hallii* | CL8.1 |
| Chr, chromosome; Peak, cM (centimorgan) position of the QTL peak; LOD, logarithm of odds; % var, present of variance explained; SE, one standard error; PC1, principal component 1; PC2, principal component 2; PC3, principal component 3; Epi, epistasis. | | | | | | | | | |
